# Hydrogen peroxide inhibition of bicupin oxalate oxidase

**DOI:** 10.1371/journal.pone.0177164

**Published:** 2017-05-09

**Authors:** John M. Goodwin, Hassan Rana, Joan Ndungu, Gaurab Chakrabarti, Ellen W. Moomaw

**Affiliations:** 1 Department of Chemistry and Biochemistry, Kennesaw State University, Kennesaw, GA, United States of America; 2 Department of Pharmacology, Oncology and Radiation Oncology, University of Texas Southwestern Medical Center, Dallas, TX, United States of America; 3 Laboratory of Molecular Stress Responses, University of Texas Southwestern Medical Center, Dallas, TX, United States of America; 4 Simmons Comprehensive Cancer Center, University of Texas Southwestern Medical Center, Dallas, TX, United States of America; National Research Council, ITALY

## Abstract

Oxalate oxidase is a manganese containing enzyme that catalyzes the oxidation of oxalate to carbon dioxide in a reaction that is coupled with the reduction of oxygen to hydrogen peroxide. Oxalate oxidase from *Ceriporiopsis subvermispora* (CsOxOx) is the first fungal and bicupin enzyme identified that catalyzes this reaction. Potential applications of oxalate oxidase for use in pancreatic cancer treatment, to prevent scaling in paper pulping, and in biofuel cells have highlighted the need to understand the extent of the hydrogen peroxide inhibition of the CsOxOx catalyzed oxidation of oxalate. We apply a membrane inlet mass spectrometry (MIMS) assay to directly measure initial rates of carbon dioxide formation and oxygen consumption in the presence and absence of hydrogen peroxide. This work demonstrates that hydrogen peroxide is both a reversible noncompetitive inhibitor of the CsOxOx catalyzed oxidation of oxalate and an irreversible inactivator. The build-up of the turnover-generated hydrogen peroxide product leads to the inactivation of the enzyme. The introduction of catalase to reaction mixtures protects the enzyme from inactivation allowing reactions to proceed to completion. Circular dichroism spectra indicate that no changes in global protein structure take place in the presence of hydrogen peroxide. Additionally, we show that the CsOxOx catalyzed reaction with the three carbon substrate mesoxalate consumes oxygen which is in contrast to previous proposals that it catalyzed a non-oxidative decarboxylation with this substrate.

## Introduction

Oxalate oxidase (OxOx, E.C. 1.2.3.4) catalyzes the cleavage of the carbon-carbon bond of oxalate to yield two moles of carbon dioxide as molecular oxygen is reduced to hydrogen peroxide [1]. Oxalate oxidase activity has been identified in numerous plant species including wheat [2], barley [3–5], sorghum [6, 7], rice [8], and beet [9, 10] where it participates in the defense against pathogens and in signaling [11]. As plant OxOx enzymes possess a single manganese ion within a single cupin (β-barrel), they are structurally classified as monocupins [12–15]. Sequence analysis indicates that oxalate oxidase from *Ceriporiopsis subvermispora* (CsOxOx) is the first manganese-containing bicupin enzyme characterized that catalyzes this reaction [16]. There exists a 49% sequence identity with the bicupin microbial oxalate decarboxylases (OxDC). OxDC catalyzes the carbon-carbon bond cleavage of oxalate to yield carbon dioxide and formate in a reaction in which there is no net oxidation or reduction [17].

Recent interest in oxalate oxidase for its potential applications to mediate the enzymatic degradation of oxalate for the prevention of scaling in the paper pulping industry [18] and as a component of enzymatic biofuel cells [19, 20] highlight the need to understand its product inhibition by hydrogen peroxide. Additionally, it has been noted that pancreatic cancer cells have increased concentrations of ascorbate derived oxalic acid compared to normal cells [21] and recent work exploring glutamine and ascorbate metabolism in pancreatic cancer [22, 23] provide a rationale to determine if hydrogen peroxide producing oxidases introduced into pancreatic cancers would have a cytotoxic effect. The only available information on the effects of hydrogen peroxide on oxalate oxidase was a survey of the effects of a variety of compounds found in bleaching filtrates (pulping) on the enzyme [24]. It was reported that hydrogen peroxide is well tolerated by barley oxalate oxidase up to 1 mM but that at 20 mM H_2_O_2_ only 30% of the initial enzymatic activity remained. The fact that OxOx is typically assayed using a continuous spectrophotometric assay in which H_2_O_2_ production is coupled to the horseradish peroxidase (HRP) catalyzed oxidation of 2,2’-azinobis-(3-ethylbenzthiazoline-6-sulphonic acid) (ABTS) [5, 25] has confounded previous efforts to address this question. In this study, we apply a membrane inlet mass spectrometry (MIMS) assay for OxOx [26] to directly measure initial rates of carbon dioxide formation and oxygen consumption in the presence and absence of hydrogen peroxide. MIMS uses a semipermeable membrane as an inlet to a mass spectrometer for the measurement of the concentration of small uncharged molecules in solution. The MIMS method of measuring oxalate oxidase activity involves continuous, real-time direct detection of oxygen consumption and carbon dioxide production from the ion currents of their respective mass peaks. ^13^C_2_-oxalate was used to allow for accurate detection of ^13^CO_2_ (m/z 45) despite the presence of adventitious ^12^CO_2_ [26].

Despite much effort, the chemistry that oxalate degrading enzymes catalyze is not fully understood. Recent investigations have informed a number of mechanistic proposals for the degradation of oxalate by OxOx and OxDC. Common features of these proposals include the binding of oxalate directly to Mn(II), the formation of Mn(III), and a radical intermediate species [27–31]. A manganese-bound formyl radical results from a reversible proton-coupled electron transfer. EPR spin-trapping experiments support the existence of an oxalate-derived radical species formed during turnover [25, 32–34] that is common to both the OxOx and OxDC mechanisms. OxOx is proposed to go through a percarbonate intermediate species before the second mole of carbon dioxide is released. In OxDC from *Bacillus subtilis*, an active site glutamic acid is proposed to protonate the manganese-bound formyl radical before formate is released [27]. Initial rate measurements in the presence of hydrogen peroxide indicate that OxDC is not inhibited by hydrogen peroxide [35]. While it has been reported that the Mn(II) EPR signal of barley OxOx is not significantly altered in the presence of H_2_O_2_ [36], a detailed consideration of the hydrogen peroxide inhibition of these enzymes is absent from the literature.

In this work, we apply the MIMS direct measurement of carbon dioxide formation and oxygen consumption to demonstrate that hydrogen peroxide is both a reversible noncompetitive inhibitor of the CsOxOx catalyzed oxidation of oxalate and an irreversible inactivator. HPLC analysis of reactants and products suggests that the build-up of turnover-generated hydrogen peroxide leads to the inactivation of CsOxOx. The introduction of catalase to reaction mixtures protects the enzyme from inactivation thus allowing reactions to proceed to completion. Circular dichroism spectra indicate that no changes to global protein structure take place in the presence of hydrogen peroxide. Furthermore, we show that the CsOxOx catalyzed reaction with the three carbon substrate mesoxalate consumes oxygen which is in contrast to previous proposals that it catalyzed a non-oxidative decarboxylation.

## Materials and methods

Recombinant oxalate oxidase from *Ceriporiopsis subvermispora* was expressed and purified as a secreted soluble protein using a *Pichia pastoris* expression system (Invitrogen) as previously described [25]. ^13^C_2_-oxalate and ^13^C_3_-glycerol were purchased from Cambridge Isotope Laboratories. Reagents were of the highest purity available and were purchased from either Sigma-Aldrich or Fisher Scientific unless otherwise stated. A modified Lowry assay (Pierce) was used to determine protein concentration using bovine serum albumin as a standard [37]. NMR spectra were recorded on a Bruker DPX 300.

### Membrane inlet mass spectrometry (MIMS)

A Hiden Analytical HPR40 membrane inlet mass spectrometer was used with a newly offered probe from Hiden Analytical designed to match that described in work from the Silverman laboratory [38–40]. The inlet probe comprised a length of tubular Silastic membrane (1.96 mm OD and 1.47 mm ID) sealed at one end by a ruby/sapphire ball (2 mm) and attached at the other end to a piece of quartz tubing (120mm length, 2.3 mm ID, and 6.35 mm OD). A stainless steel membrane support spring (316SS) was used to support the tubular Silastic membrane at running vacuum pressures. The length of membrane from the ruby/sapphire ball to the beginning of the glass tubing was 3.3 mm. For some measurements, the quartz tubing was connected to a 76.3 cm length of Teflon tubing exiting into the main vacuum chamber of a Hiden Analytical HPR-40 DSA Membrane Inlet Mass Spectrometer. When measuring oxygen consumption, the quartz tubing was attached to an elbow joint that exited directly into the main vacuum chamber.

The membrane inlet mass spectrometer was calibrated by measuring solutions of known CO_2_ concentration prepared by adding solutions of K_2_CO_3_/KHCO_3_ (pH 10.2) into the reaction vessel containing 50 mM acetic acid. The ion current at m/z 44 was recorded and plotted versus CO_2_ concentration (Figure A in S1 File). The sensitivity of the measurements was directly proportional to the stir rate so that standard curves were prepared for each experimental condition. Similarly, a standard curve for O_2_ is prepared by recording the average ion currents at m/z 32 in solutions of different O_2_ concentrations prepared by dilution/mixing of O_2_, or air saturated reaction buffer at 25°C (not shown).

### Steady-state kinetic assays

In order to distinguish the CO_2_ generated by CsOxOx from CO_2_ dissolved in the reaction mixtures, doubly ^13^C labelled oxalate was employed. We measured the production of CO_2_ through the m/z 45 peak (^13^CO_2_) and the consumption of O_2_ through the m/z peak 32. The 2.0 mL reaction typically contained 50 mM succinate, pH 4.0, 10 mM ^13^C_2_–oxalate, pH 4.0 and was initiated at 5 minutes by the addition of enzyme to a final concentration of 0.10 μM. The response time of the instrument is about 6 seconds. To convert the measured ion currents into reactant and product concentrations and rates, the spectrometer was calibrated for CO_2_ and O_2_ as described above. A typical experiment is shown in Supporting Information Figure B in S1 File. Measurements made using the MIMS assay are in good agreement with those from the HRP coupled spectrophotometric assay [26]. The m/z 45 of reaction mixtures were measured until the rate of baseline decay was less than 5 x 10^−15^ torr per millisecond over two minutes. Upon enzyme addition, the initial rate was subtracted from the baseline. All standard curve and kinetic measurements were made in duplicate. The initial reaction rates at specific substrate and enzyme concentrations were fit to the Michaelis-Menten formula (Eq 1) using the software KaleidaGraph to determine the kinetic parameters *V*_*max*_ and *K*_*M*_.

$$v=\frac{V_{max}[S]}{K_{M}+[S]}$$ (1)

In order to demonstrate the reversible nature of the hydrogen peroxide inhibition of the CsOxOx catalyzed oxidation of oxalate, kinetic parameters were determined from initial rate measurements made in initial concentrations of 2 mM, 4 mM, and 10 mM hydrogen peroxide. To determine hydrogen peroxide kinetic constants of inhibition (*K*_*I app*_ and α*K*_*I app*_ values), kinetic parameters were determined from initial rate measurements made in initial concentrations of 10 mM, 20 mM, and 40 mM hydrogen peroxide. The hydrogen peroxide inhibition kinetic model was approximated to a unireactant noncompetitive system as it is assumed that oxygen binding occurs after oxalate binding and that the oxygen concentration is large enough to assume the binding of oxygen is irreversible. This is reasonable since the *K*_*M*_ for dioxygen was reported to be less than 70 μM [26] and the concentration of oxygen in air saturated solution is 256 μM at 25°C. The hydrogen peroxide kinetic constants *K*_*I app*_ and α*K*_*I app*_ where determined from a series of Lineweaver-Burk plots and the respective secondary plots of slopes or x-intercepts of the Lineweaver-Burk plots versus hydrogen peroxide concentration. The value *K*_*I app*_ was estimated from the x-intercept of the slope secondary plot, the value α*K*_*I app*_ was estimated from the x-intercept of the x-intercept secondary plot. The value α was estimated by two methods. The first estimation was done by taking the ratio of α*K*_*I app*_ to *K*_*I app*_ (Eq 2).

$$\alpha =\frac{{\alpha K}_{I\;app}}{K_{I\;app}}$$ (2)

The second estimation was done by determining the inverse velocity at the point around which the Lineweaver-Burk plots constellate. The inverse initial velocity at the point of intersection (1/v_int_) and the V_max_ values were then used to find α by Eq 3.

$$\alpha =\frac{1}{1-\frac{1}{v_{int}}V_{max}}$$ (3)

In order to distinguish reversible inhibition from irreversible inactivation, a plot was constructed in which the *V*_*max app*_ (in total U) was determined using the MIMS assay as previously described for assay mixtures containing 0 and 10 mM hydrogen peroxide [41]. *V*_*max app*_ values were obtained by measuring initial rates at five concentrations of potassium oxalate, pH 4.0 ranging from below 0.1 K_M_ to 10 K_M_ for oxalate (0.2 mM, 0.5 mM, 1.0 mM, 5.0 mM, and 10.0 mM final oxalate concentrations) at total enzyme concentrations of 104.5, 69.7, 34.8, and 17.4 nM (measured in duplicate). *V*_*max* app_ values were plotted against total enzyme concentration. To test that the observed decreased initial rates were the result of inhibition and not inactivation, 772 nM CsOxOx was pre-incubated for two hours at 25°C with 10 mM hydrogen peroxide present. The enzyme was then assayed at a final enzyme concentration of 77.2 nM in final concentrations of 1 mM and 10 mM hydrogen peroxide.

In order to explore the inactivation of CsOxOx during turnover, 772 nM enzyme was incubated at 25°C with 10 mM potassium oxalate in 50 mM sodium succinate, pH 4.0 in the presence and absence of 1.0 mg/mL bovine catalase [42]. To test the stability of the enzyme, 772 nM enzyme was incubated also at 25°C without oxalate present. Aliquots were removed at time 0, 2 hours, and 24 hours and assayed to determine initial rates under saturating substrate conditions (>10 *K*_*M*_) as described in the MIMS assay. Total enzyme concentrations of 128.8, 103.0, 77.2, and 51.5 nM were measured in duplicate. Rates under these conditions were plotted against total enzyme concentration for the 0 and 2 hour time points.

### Horseradish peroxidase coupled steady-state kinetic assay

A continuous spectrophotometric oxalate oxidase activity in which the production of H_2_O_2_ is coupled to the horseradish peroxidase (HRP) catalyzed oxidation of 2,2’-azinobis-(3-ethylbenzthiazoline-6-sulphonic acid) (ABTS) was also used to measure the rate of hydrogen peroxide formation [5]. Reaction mixtures contained 25 U HRP, 5 mM ABTS, 50 mM potassium oxalate (or mesoxalate), and CsOxOx dissolved in sodium succinate, pH 4.0 (total volume 1.0 mL). An extinction coefficient of 10,000 M^-1^ cm^-1^ (at 650 nm) for the ABTS radical product was assumed in these experiments. Samples omitting HRP served as a control in order to distinguish between H_2_O_2_ production and any oxalate-dependent dye oxidation activity by CsOxOx. Measurements were made at specific substrate and enzyme concentrations in duplicate, and data were analyzed to obtain the values of *V*_*max app*_ and *K*_*M app*_ by standard computer-based methods [43].

### HPLC analysis of the products of the CsOxOx catalyzed oxidation of oxalate

HPLC analyses were carried out using a 300mm x 7.8 mm Aminex HPX-87H ion exchange column (Bio-Rad) attached to a Dionex HPLC system fitted with a 25 μL sample loop and UV/Vis detection at 230 nm. The mobile phase was 4.0 mM sulfuric acid and all separations were made at 25°C in isocratic and isothermal 40 minute runs with flow rates of 0.6 mL/min. The composition of samples was determined by comparison of retention times of sample peaks with those of commercially available mesoxalate, oxalate, and glyoxylate. Reactions containing 50 mM sodium succinate at pH 4.0, approximately 5 μg/mL of CsOxOx and 10 mM of potassium oxalate were incubated for 24 hours at 25°C. At times 0 minutes, 20 minutes, 1 hour, 2 hours, and 24 hours, 50 μL aliquots were removed from the reaction mixture and injected into the HPLC and assayed using the MIMS CsOxOx assay. Identical reactions containing 0.5 mg/mL of bovine catalase were also performed and analyzed.

### Circular Dichroism (CD) studies

All Circular Dichroism (CD) experiments were performed using a JASCO J-1500 Spectropolarimeter (JASCO Inc., Tokyo, Japan) using a 0.1-cm path length cell. All samples were exchanged into 25 mM potassium phosphate (pH 7.0). Analysis of CD spectra was performed using Spectra Analysis (JASCO Inc., Tokyo, Japan). To test the effect of hydrogen peroxide on global protein structure of CsOxOx, spectra were recorded of a 1.0 mg/mL solution at 4 mM, 8 mM, 12 mM, 16 mM, and 20 mM hydrogen peroxide at 25°C. In order to observe the effects of thermal denaturation on the secondary structural elements of CsOxOx, spectra were recorded of a 0.68 mg/mL solution at increasing temperatures (10°C to 90°C, with 10°C increments). Each spectrum was taken after ten minutes of incubation at each temperature. A Teflon stopper was used to retard evaporation. The scan rate, time constant and numbers of scans were 10 nm/min, 2 s, and 3, respectively. Each spectrum was an accumulation of five scans. A blank spectrum was performed with buffer and subtracted from the spectra.

## Results and discussion

### Initial rate measurements reveal that hydrogen peroxide is a reversible noncompetitive inhibitor of the CsOxOx catalyzed oxidation of oxalate

**Fig 1:**
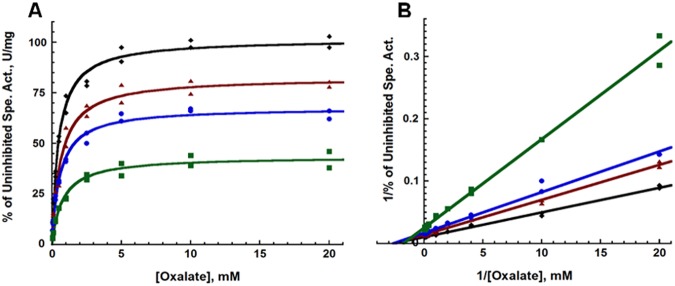
Michaelis-Menten (A) and Lineweaver-Burk (B) plots of the initial rates of oxalate oxidation by CsOxOx demonstrating the effects of varying initial hydrogen peroxide concentrations: black, no H_2_O_2_; red, 2 mM H_2_O_2_; blue, 4 mM H_2_O_2_; green, 10 mM H_2_O_2_.

Product inhibition is a special case of inhibition in which the inhibitor is also a product of the enzyme catalyzed reaction. Typically kinetic studies of enzymatic activity are done under conditions at which the amount of product present during the reaction is effectively zero. This removes the effects of the reverse reaction forming reactants as well as those effects observed by the binding of product to enzyme thus simplifying the velocity equation dramatically, and, in the case of a simple unireactant system, producing the well-known Michaelis-Menten equation (Eq 1). Introducing non-zero initial concentrations of product reintroduces the product dependent terms of the velocity equation and often allows one to differentiate between two similar potential kinetic models. The Michaelis-Menton and Lineweaver-Burk plots for varying concentrations of hydrogen peroxide are shown in Fig 1A and 1B, respectively. The percent of the uninhibited specific activity and *K*_*M*_ values for the CsOxOx catalyzed oxidation of oxalate in the presence and absence of hydrogen peroxide measured by MIMS is presented in Table 1. Initial concentrations of hydrogen peroxide reduce the apparent maximum velocity of catalysis with only mild perturbations (a 60% increase) of the observed *K*_*M* app_ values. These data suggest that hydrogen peroxide behaves as a noncompetitive inhibitor of CsOxOx. Noncompetitive inhibitors may bind to the enzyme form that the substrate binds and a different enzyme species [44, 45]. In the classic noncompetitive case, the equilibrium constants for the binding of inhibitor with enzyme alone (*K*_*I*_) and inhibitor with another enzyme species (α*K*_*I*_, described further below) are equal. When the two equilibrium constants are different, traditional inhibition nomenclature becomes ambiguous with this case often defined as a “mixed-type” inhibitor and not a noncompetitive inhibitor. However, Cleland states that since there is no *a priori* reason that the equilibrium constants should be equal, mixed inhibition should be referred to as noncompetitive inhibition [44]. By this definition, a noncompetitive inhibitor will reduce the apparent maximal velocity, but can reduce, increase or have no effect on the apparent *K*_*M*_ value. All models consistent with this observation have hydrogen peroxide reversibly binding both CsOxOx alone and another CsOxOx complex.

**Table 1:** Percent of the uninhibited specific activity and K_M_ values for the CsOxOx catalyzed oxidation of oxalate in the presence and absence of hydrogen peroxide measured by MIMS.

Substrate ± H_2_O_2_	% of Uninhibited Spe. Act., U/mg	*K*_M app,_ mM^a^
**Oxalate**	100	0.47 ± 0.03
**+ 2 mM H**_**2**_**O**_**2**_	81.3	0.47 ± 0.03
**+ 4 mM H**_**2**_**O**_**2**_	66.5	0.56 ± 0.04
**+ 10 mMH**_**2**_**O**_**2**_	42.9	0.76 ± 0.08

^a^Uncertainties represent standard errors in the fit to the Michaelis–Menten expression.

**Fig 2:**
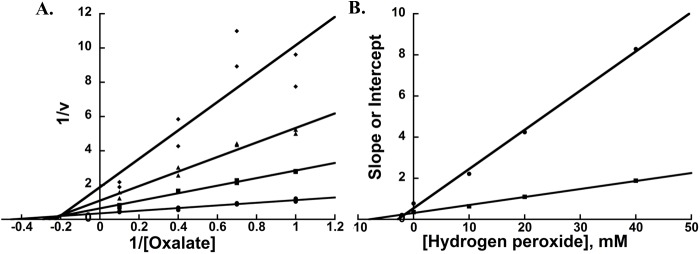
(A) Lineweaver-Burk plots of initial oxalate oxidase activity versus oxalate concentration for CsOxOx in varying H_2_O_2_ concentrations: circles, no H_2_O_2_; squares, 10 mM H_2_O_2_; diamonds, 20 mM H_2_O_2_; triangles, 40 mM H_2_O_2_. (B) Secondary plots of the slopes and intercepts from of the reciprocal plot linear best fit lines (Fig 2A) versus hydrogen peroxide concentration; circles, slopes; squares, intercepts.

To assign *K*_*I app*_ and α*K*_*I app*_ values for the noncompetitive inhibition observed in Fig 1A and 1B, experiments at substrate concentrations resulting in Lineweaver-Burk plots of equidistance and equal weight points were carried out in a larger range of hydrogen peroxide concentrations. The resulting plots of reciprocal initial oxalate oxidase activity versus reciprocal oxalate concentration for CsOxOx are shown in Fig 2A. Fig 2B shows the secondary plots of the slopes and intercepts of the reciprocal plot linear best fit lines versus hydrogen peroxide concentration. The α*K*_*I app*_ and *K*_*I app*_ values are estimated to be 7.91 ± 1.12 and 2.84 ± 1.06 from the x-intercepts secondary plots [44]. The slope and intercept secondary plots are linear (R^2^ values greater than 0.99) which suggests that the hydrogen peroxide inhibition of CsOxOx is complete (no product is released from the inhibitor and the different enzyme species) and not partial (up to 40 mM hydrogen peroxide). From these data, α was estimated to be 2.79 ± 1.12. A way to mathematically check this value is to use the regression lines from the reciprocal plots (Fig 2B) [44]. The lines intersect at the point -0.207±0.011 on the x-axis and 0.195±0.042 on the y-axis, which corresponds to an α value of 2.25 ± 0.59. The values for the α constant determined using two different the methods are in good agreement.

**Fig 3:**
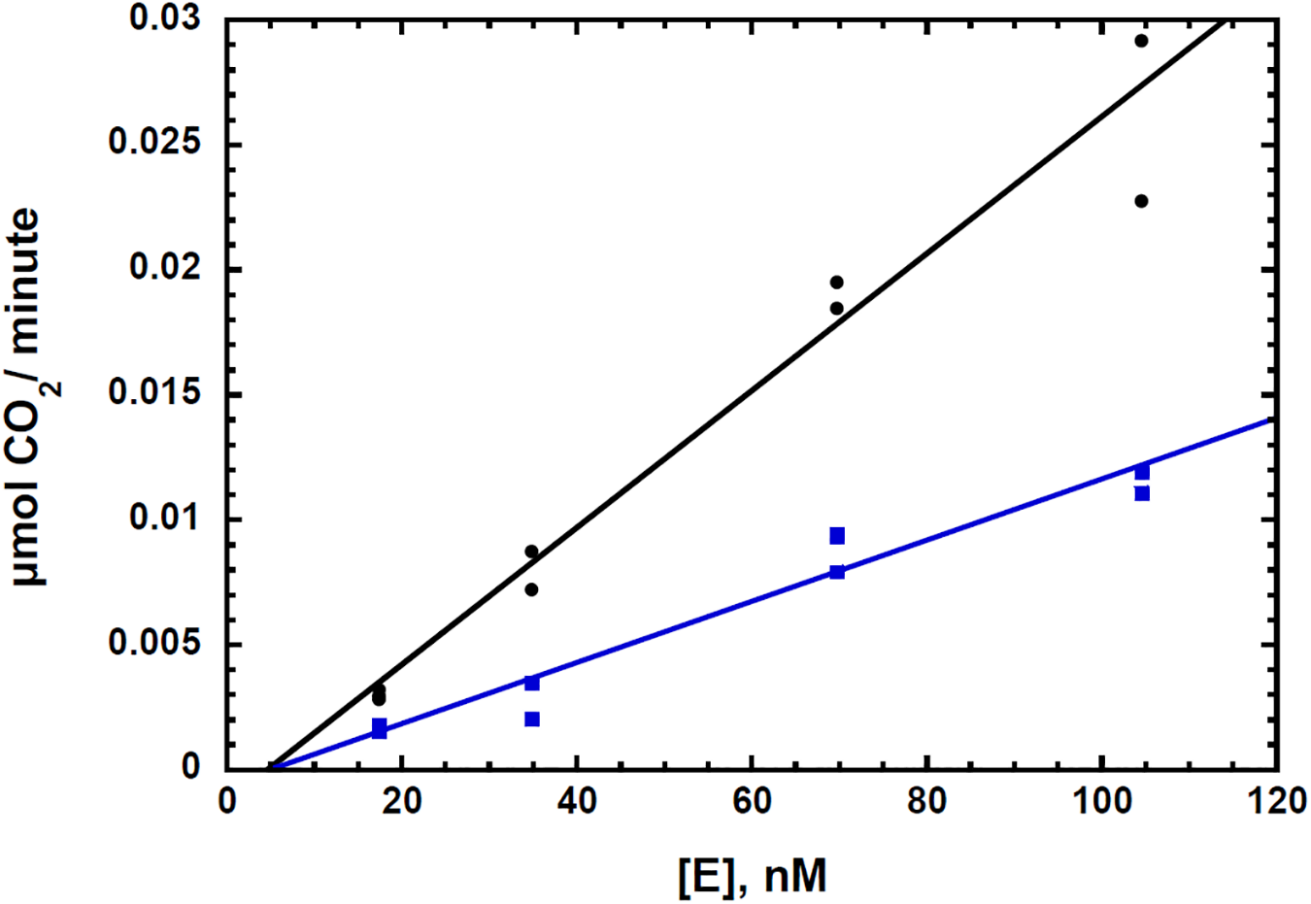
Plot of V_max app_ versus [E]_t_ of the CsOxOx catalyzed oxidation of oxalate with (blue, 10 mM) and without (black) hydrogen peroxide present. Each point represents a *V*_*max* app_ determination at five concentrations of oxalate (0.2, 0.5, 1.0, 5.0, and 10.0 mM).

Reversible noncompetitive inhibition and irreversible inhibition may be distinguished by plotting *V*_*max app*_ data as a function of [E]_t_, where [E]_t_ represents the total units of enzyme activity added to the assay [41]. These data for the hydrogen peroxide inhibition of the CsOxOx catalyzed oxidation of oxalate are shown in Fig 3. The rate of product formation is equal to the product of the enzyme concentration and a kinetic factor which is a function of all substrate, product activator, and inhibitor concentrations. The reaction rate of an enzyme catalyzed reaction is, therefore, a linear function of the enzyme concentration with a *y-*intercept of 0 and a slope of *f([S]*, *[P]*, *[A]*, *[I])* [42]. A non-zero *x-*intercept implies that the concentration of enzyme has been changed with the difference of the *x*-intercept from zero defining the concentration of active enzyme removed or added. Reversible inhibitors by definition do not permanently inactivate the enzyme and therefore can only affect the slope of the line but not its *x*-intercept. In Fig 3 the curves with and without initial concentrations of hydrogen peroxide present do not appear to have different *x*-intercepts. The x-intercept for the plot without inhibitor present was 7 nM ± 8 nM and the x-intercept for the plot with inhibitor present was 8 nM ± 9 nM. These x-intercepts from both linear fits are not significantly different from each other and they are not significantly different from zero (one standard deviation from the x-intercept for both lines includes zero) suggesting that CsOxOx is not inactivated upon addition of hydrogen peroxide within the time frame of the kinetic assay. Since the curve with hydrogen peroxide present has a smaller slope and goes through the approximate origin, these data are consistent with hydrogen peroxide being a reversible noncompetitive inhibitor of the CsOxOx catalyzed oxidation of oxalate. In the case of an irreversible inhibitor, the slope of the line with inhibitor present would have the same slope and intersect the *x-*axis at a position equal to the amount of enzyme that is irreversibly inactivated [41, 42]. It is important to note that these data (Fig 3) show that the concentration of irreversibly inactivated enzyme is negligible under initial rate conditions.

### Turnover-generated hydrogen peroxide leads to inactivation of CsOxOx

**Fig 4:**
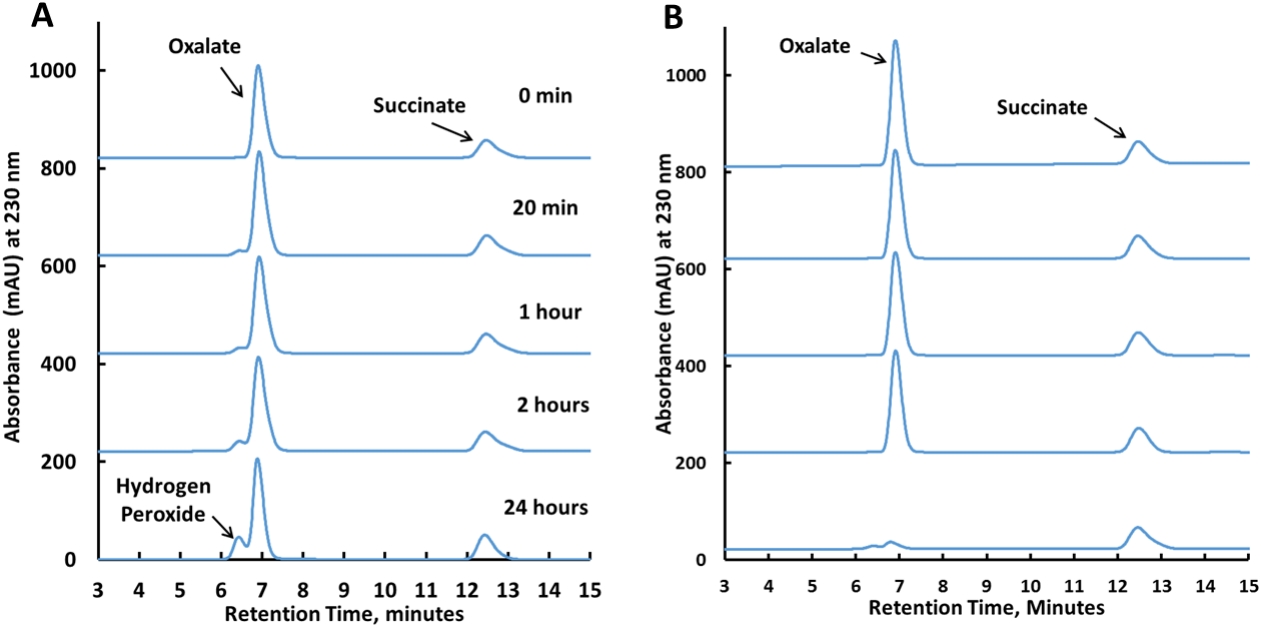
HPLC analysis of the reaction products of the CsOxOx catalyzed of oxalate. An aliquot of the incubation reaction without (A) or with (B) recombinant bovine catalase was analyzed at initiation, 20 minutes, 1 hour, 2 hours, and 24 hours.

HPLC chromatograms monitoring the amounts of hydrogen peroxide and oxalate at discrete time points are shown in Fig 4A (no catalase present) and 4B (catalase present). Hydrogen peroxide, oxalate and succinate standards eluted at 6.3 minutes, 6.9 minutes, and 12.4 minutes, respectively and informed the peak assignments shown. In the absence of catalase the amount of oxalate remaining after 24 hours was approximately 85% of the initial amount (10 mM) and the appearance of an accumulation of hydrogen peroxide was visible in the chromatogram. The proximity of the hydrogen peroxide peak and the oxalate peak confounded precise integration of their respective areas. After 24 hours, an aliquot of the sample without catalase had no detectable activity in the MIMS assay suggesting that the CsOxOx present had become inactivated by the build-up of turnover-generated hydrogen peroxide. Further, the addition of catalase to the inactivated enzyme does not return any active CsOxOx from the observed inactivation. These results suggest that hydrogen peroxide is having at least two separate effects on CsOxOx. First, hydrogen peroxide behaves as a reversible noncompetitive inhibitor of the initial reaction velocity as described above. Separately, hydrogen peroxide also appears to be involved in the slow onset inactivation of the CsOxOx enzyme observable minutes to hours after initial enzyme turnover.

**Fig 5:**
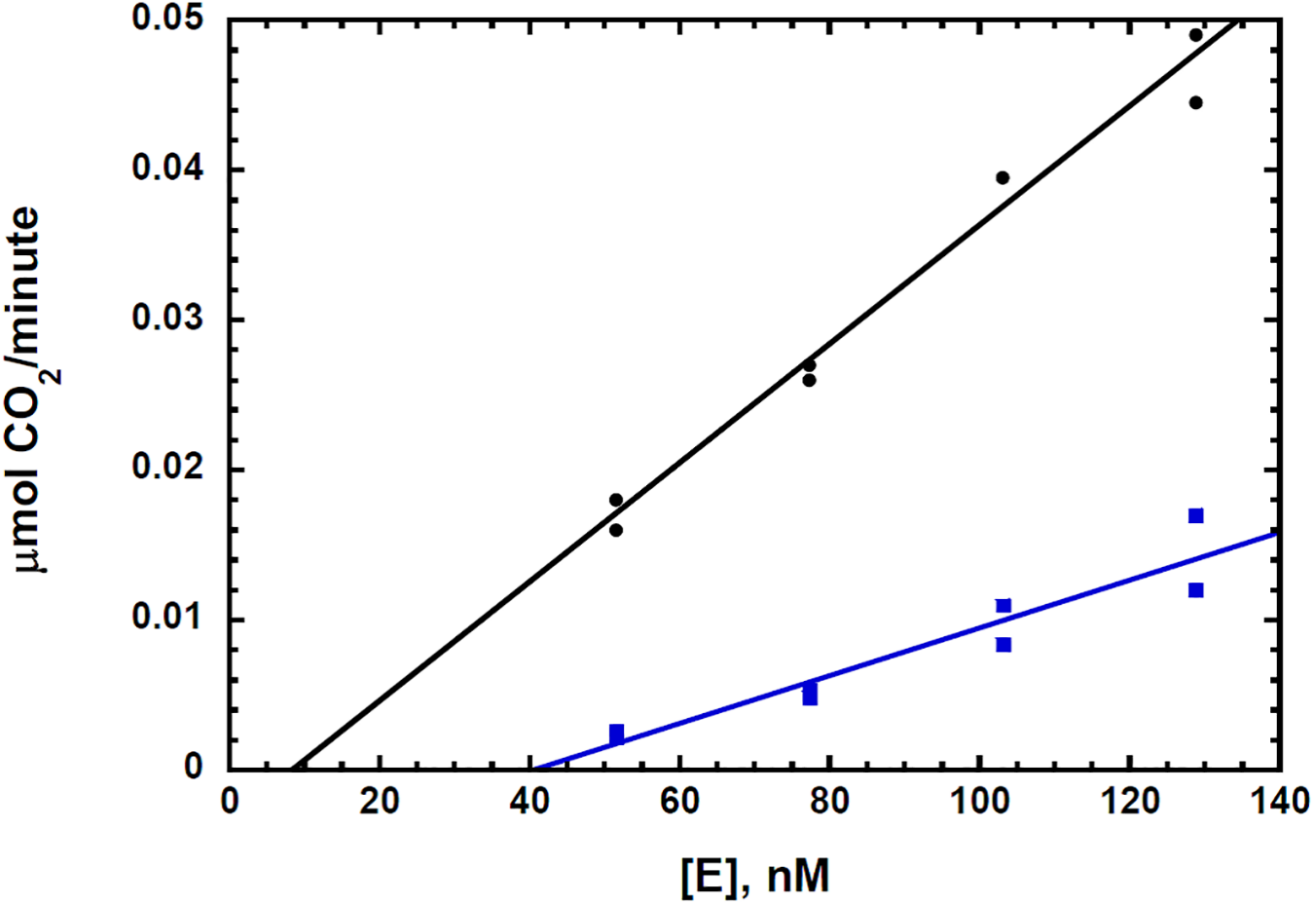
Plot of rates under saturating oxalate (>10 K_M_) versus [E]_t_ of the CsOxOx catalyzed oxidation of oxalate after no prior turnover (black) and prior incubation with oxalate (blue, 10 mM, 2 hours).

In order to observe the effects of turnover on CsOxOx, a plot of reaction velocities after 0 and 2 hours of turnover is shown in Fig 5. Similar to the data in Fig 3, the slope of the turnover “treated” (two hours of turnover in the presence of 10 mM oxalate) initial rate curve is smaller than that of the “untreated” (no prior turnover) control. This suggests that a perturbation of the kinetic function is at least partially due to the build-up of the hydrogen peroxide product during the two hours of turnover. In Fig 5, however, the x-intercept of the turnover treated curve is different from the control curve. This observation is consistent with inactivation of CsOxOx over the two hour time period [41, 42]. The inactivation of the enzyme is only seen when the enzyme is pre-incubated with oxalate. Pre-incubation in buffer or buffer and hydrogen peroxide showed no inactivation. Specifically, CsOxOx preincubated with 10 mM hydrogen peroxide for two hours then assayed at final concentrations of 1 mM and 10 mM hydrogen peroxide resulted in measured initial rates that were commensurate with those expected from the inhibition studies described above (91.4% and 44.3% of the uninhibited specific activity, respectively). Enzyme pre-incubated with 10 mM potassium oxalate possessed less than 20 percent of the original enzyme activity after two hours (data not shown) and no activity was detectable after 24 hours. Table 2 shows the results of MIMS measurements of samples with no prior turnover, turnover in the absence and in the presence of 1.0 mg/mL bovine catalase as described in the Materials and Methods section. The enzyme was reasonably stable under the conditions of the experiment with 73 percent of original enzyme activity remaining after incubation at 25°C for 24 hours. When catalase is present, however, approximately 80 percent of the enzyme activity remains after 24 hours. These data suggest that hydrogen peroxide or a derivative there of has a key role in the turnover-dependent inactivation of CsOxOx.

**Table 2:** MIMS measurements of CsOxOx with no prior turnover, turnover in the absence and in the presence of 1.0 mg/mL bovine catalase.

Time, hours	No prior turnover, U/min^a^^,^^b^	Turnover Treated, U/min	Turnover treated plus catalase, U/min
**0**	0.026 ± 0.001	0.026 ± 0.001	0.030 ± 0.001
**24**	0.019 ± 0.001	nd^c^	0.024 ± 0.003

^a^ error reported is the standard deviation of duplicates.

^b^ a final enzyme concentration of 77.2 nM was used in all determinations.

^c^ nd is not detectable under the conditions of the assay.

It is not immediately apparent how the presence of turnover-generated hydrogen peroxide inactivates CsOxOx. One proposed mechanism for barley oxalate oxidase catalysis identifies the fraction of enzyme containing Mn^3+^ as the active form of the enzyme [28]. In this proposal, turnover is initiated after oxalate binds to the metal center and an electron is transferred from the oxalate ligand to a Mn^2+^ ion. The loss of an electron facilitates the decarboxylation of the oxalate ligand leaving a manganese bound carbon dioxide radical anion which is proposed to react with diatomic oxygen to produce carbon dioxide and a hydroperoxyl radical. Spectroscopic studies consistent with the persistence of the hydroperoxyl radical species being critical to enzyme turnover support this proposal. Whitaker *et al* have hypothesized that the hydroperoxyl radical directly reoxidizes the Mn^2+^ ion, reactivating the enzyme to the Mn^3+^ form for further turnover. Loss of the hydroperoxyl radical would thus prevent the reoxidation of the metal center rendering it inactive [28]. A similar mechanism of inactivation of OxDC was put forth to describe the EPR observation of a spin trapped carbon dioxide radical species suggesting a “leaky” active site from which the carbon dioxide radical can dissociate and enter bulk solution. Again, it was proposed that in the absence of an electron sink at the active site, the inactivation of the enzyme would occur after carbon dioxide radical dissociation [33]. It is important to note that the Whitaker *et al* mechanism and observations for monocupin barley oxalate oxidase may not be accurate for CsOxOx. One significant difference is that the barley enzyme is inactivated under anaerobic condition in the presence of oxalate and remains so even after the reintroduction of oxygen. In contrast, CsOxOx, while inactive under anaerobic conditions in the presence of oxalate, regains activity upon reintroduction of oxygen (data not shown). Another significant difference is that the presence of catalase accelerated enzyme inactivation of the barley enzyme while catalase protects against inactivation in CsOxOx. These observations are not consistent with a mechanism for CsOxOx utilizing a hydroperoxyl radical alone for the regeneration of catalytically competent enzyme, and supports the role of the Mn(II) in the reductive activation of diatomic oxygen. Our results suggest a completely different method of inactivation. We observe that enzyme inactivation occurs during CsOxOx turnover and only in the presence of hydrogen peroxide. This suggests that hydrogen peroxide is reacting irreversibly with a turnover intermediate rendering the enzyme inactive reminiscent of the hydrogen peroxide inactivation of catalase [46].

MIMS measurements reveal approximately 1.3 moles of CO_2_ is produced per one mole of O_2_ consumed. When this ratio is plotted as a function of oxalate (from 0.5 to 10 mM) in the reaction mixture (Figure C in S1 File), the result is essentially a horizontal line indicating that the ratio is independent of initial oxalate concentration. Furthermore, the ratio of CO_2_ produced per one mole of O_2_ consumed is independent of hydrogen peroxide concentration (in both initial rate measurements and in the case of turnover induced inactivation). Despite the application of numerous assay techniques and much interest in the stoichiometry of this reaction, this is to our knowledge the first report of the observed stoichiometric ratio of moles of CO_2_ produced per mole of O_2_ consumed of oxalate oxidase. It has been reported that for sorghum OxOx, the consumption of oxalate was directly related to the formation of hydrogen peroxide [6]. The application of Warburg manometric techniques to peroxisomal preparations from the leaves of the spinach beet enzyme were not able to establish the stoichiometry due to the small amount of oxygen consumed and carbon dioxide formed [10]. Manometric measurements of rates of the enzyme from the parasitic fungus *Tilletia controversa* were confounded by impure enzyme preparations [47]. Liquid scintillation procedures were used to relate ^14^CO_2_ formation to ^14^C-oxalate disappearance but the consumption of oxygen was not measured in those experiments [48]. Interestingly, oxalate decarboxylase from *Bacillus subtilis* (BsOxDC) has been mutated (SENS161-4DASN) to carry out the oxidation reaction and a ratio of 1.3 moles of hydrogen peroxide produced to 1 mole of the one electron oxidation of the dye ABTS has been reported [27]. Recent MIMS measurements of this mutant BsOxDC assumed a ratio of 2 moles of CO_2_ is produced per one mole of O_2_ consumed at low oxalate concentrations [29]. This assumption contributed to the conclusion that only 9% of the total number of active sites of the SENS161-4DASN mutant BsOxDC carry out the oxidation reaction with the remaining sites nonoxidatively decarboxylating oxalate.

Previously proposed mechanistic schemes all assume a 2 to one molar relationship between CO_2_ production and O_2_ consumption. That a 1.3 to one ratio is observed in all conditions tested raises the possibility that an additional oxidation may be taking place. One possible explanation could reside in the fate of the carbon dioxide radical anion formed after the first decarboxylation. As mentioned above, the spin trapping of this species in both CsOxOx and OxDC [25, 32–34], suggests that it leaks out the active site. This radical may react with dissolved oxygen from bulk solution to form a superoxide radical. Superoxide radicals have been trapped in the case of the OxDC catalyzed redox neutral decarboxylation of oxalate [33]. Since superoxide radicals (pKa of 4.88) exist primarily as hydroperoxyl radicals under our experimental conditions, escape of this species from the active site and subsequent oxidation of exogenous species, would result in the observed increased consumption of oxygen [28].

### Circular Dichroism (CD) studies of CsOxOx show no global structural changes in the presence of hydrogen peroxide

**Fig 6:**
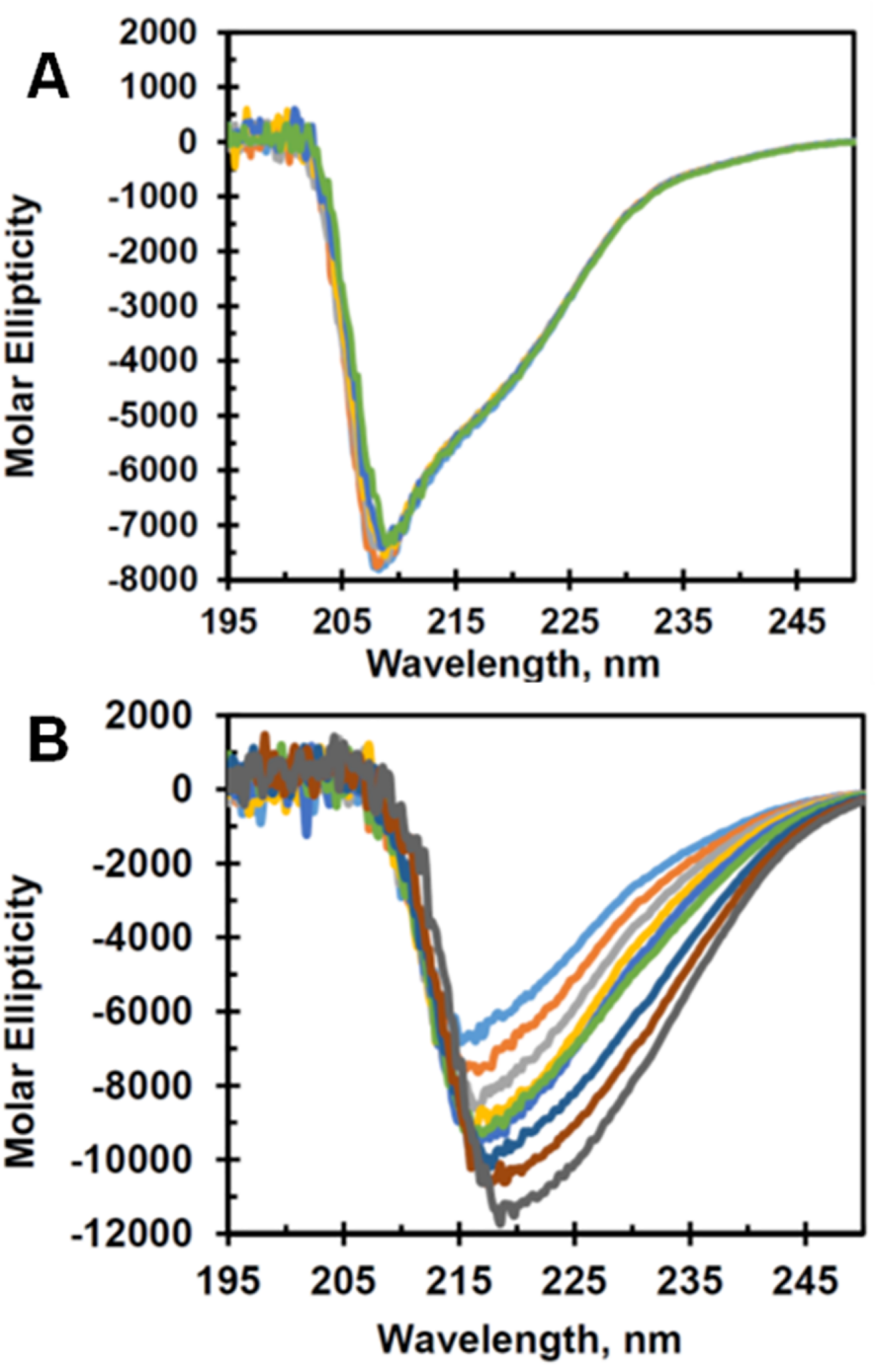
Circular dichroism spectra of CsOxOx. A. CD spectra of wild-type CsOxOx in the presence and absence of hydrogen peroxide: light blue, no H_2_O_2_; orange, 4 mM H_2_O_2_; grey, 8 mM H_2_O_2_; yellow, 12 mM H_2_O_2_; dark blue, 16 mM H_2_O_2_; green, 20 mM H_2_O_2_. B. CD spectra of .0.68mg/ml CsOxOx in 25 mM potassium phosphate (pH 7.0) at different temperatures: dark grey, 90°C; rust, 80°C; dark blue, 70°C; blue, 60°C; green, 50°C; yellow, 40°C; grey, 30°C; orange, 20°C; light blue, 10°C.

CD experiments were performed to monitor the secondary structure as a function of hydrogen peroxide concentration and temperature. Fig 6A shows that the CD spectrum for CsOxOx is unperturbed by hydrogen peroxide concentrations up to 20 mM. These data indicate that hydrogen peroxide does not affect the overall global protein structure of the enzyme. In contrast, the effect of temperature on the CD spectrum of CsOxOx is shown in Fig 6B. Spectra taken at higher temperatures exhibit a deeper minimum and a shift toward the near UV region. Typically, examples in the literature of the effect of temperature on the CD spectrum of proteins show a lesser degree of molar ellipticity upon increasing temperature [49]. There are, however, examples where the CD spectra display a greater degree of molar ellipticity as does CsOxOx [50].

### HPLC and MIMS analysis suggests that the products of the CsOxOx mediated oxidation of mesoxalate are carbon dioxide and hydrogen peroxide

**Fig 7:**
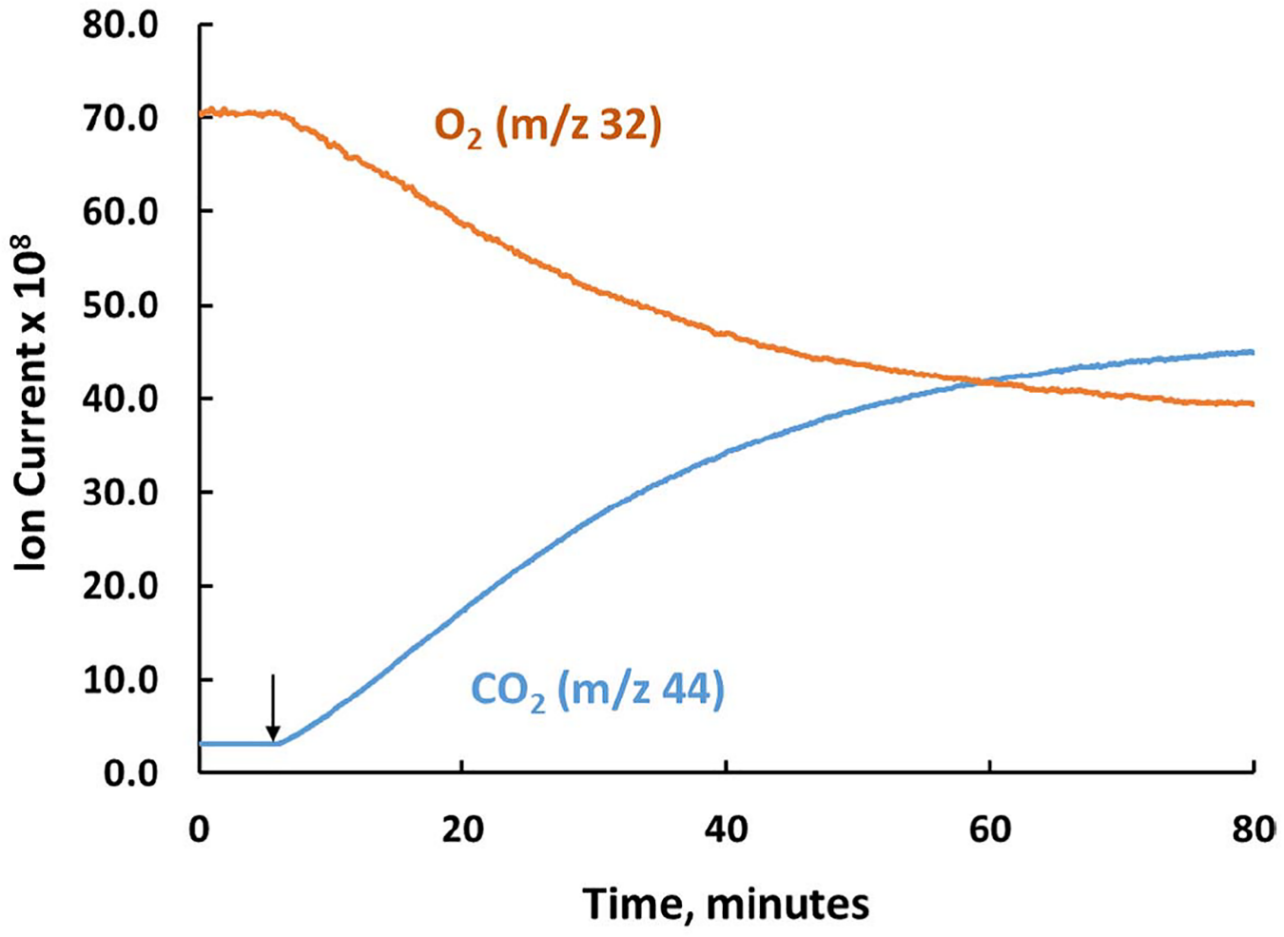
The production of CO_2_ and consumption of O_2_ (in arbitrary ion currents) from mesoxalate catalyzed by CsOxOx. The ion currents for the dissolved gases at their respective peak heights were recorded: blue, CO_2_ at m/z 44; orange, O_2_ at m/z 32. The solution contained 50 mM potassium mesoxalate and 50 mM sodium succinate buffer at pH 4.0 and 25°C. The 2 mL reaction was initiated by the addition of recombinant CsOxOx to a final concentration of 0.12 μM at 2 minutes.

We have previously used multiple injection isothermal titration calorimetry (ITC) to demonstrate that the three carbon molecule mesoxalate (oxopropanedioic acid) serves as a substrate for the CsOxOx-catalyzed reaction, with kinetic parameters comparable to that of oxalate, and to identify a number of small molecule carboxylic acid compounds that also serve as substrates for the enzyme [51]. It was previously proposed that the OxOx catalyzed reaction with mesoxalate yielded glyoxylate and carbon dioxide [19] in a reaction in which there is no net oxidation or reduction. In an effort to establish, this we carried out an HPLC analysis of the reaction products over time similar to that described above for the CsOxOx catalyzed oxidation of oxalate. No glyoxylate, however, was observed (data not shown). Furthermore, MIMS measurements using unlabeled mesoxalate resulted in rates comparable to those measured using the ITC assay and the horseradish peroxidase coupled spectrophotometric assay. A typical MIMS experiment using 10 mM mesoxalate as the substrate is shown in Fig 7. MIMS measurements show clear oxygen consumption demonstrating that CsOxOx oxidatively decarboxylates mesoxalate. From these data we propose that the products of the CsOxOx mediated oxidation of mesoxalate are carbon dioxide and hydrogen peroxide. Since mesoxalate exists primarily as the hydrated form [52], we suggest the following balanced equation: C_3_O_6_H_4_ + O_2_ → CO_2_ + C_2_O_4_H_2_ + H_2_O_2._ Here we briefly note that to explore this reaction further we attempted the preparation of ^13^C_3_-mesoxalate from ^13^C_3_-glycerol. In our hands, however, the application of a published procedure for the one pot preparation of mesoxalate through the oxidation of glycerol by TEMPO and sodium hypochlorite [53] yielded oxalate instead of the desired product. That the oxidation of ^13^C_3_-glycerol by TEMPO and sodium hypochlorite yields ^13^C_2_-oxalate is described in the Supporting Information section and ^13^C NMR spectra are shown in Figures D-F in S1 File.

In summary, potential applications of CsOxOx have underscored the need to better understand the hydrogen peroxide inhibition CsOxOx. Through the application of the MIMS direct measurement of carbon dioxide formation and oxygen consumption, we have demonstrated that hydrogen peroxide is both a reversible noncompetitive inhibitor and an irreversible inactivator of the CsOxOx catalyzed oxidation of oxalate. HPLC analysis of reactants and products indicate that the build-up of turnover-generated hydrogen peroxide leads to the inactivation of CsOxOx. The introduction of catalase to reaction mixtures protects the enzyme from inactivation thus allowing reactions to proceed to completion and has broader implications for the use of CsOxOx as a biocatalyst. Circular dichroism spectra indicate that no changes to global protein structure take place in the presence of hydrogen peroxide. The MIMS assay of the CsOxOx catalyzed reaction with the three carbon substrate mesoxalate demonstrates that oxygen is consumed which is in contrast to previous proposals that it catalyzed a non-oxidative decarboxylation.

## Supporting information

S1 File Supplementary Information. Figure A: Standard curve constructed by measuring the ion current (arbitrary scale) at m/z 44 of solutions of known CO_2_ content. Figure B: The consumption of O_2_ and production of CO _2_ (in arbitrary ion currents) during the CsOxOx catalyzed oxidation of ^13^C_2_–oxalate. Figure C: Plot of the ratio of moles CO_2_ formed per mole of oxygen consumed as a function of oxalate concentration. Text A: The oxidation of ^13^C_3_-glycerol by TEMPO and sodium hypochlorite yields ^13^C_2_-oxalate. Figure D: ^13^C NMR of product of the oxidation of ^13^C_3_-glycerol by TEMPO and sodium hypochlorite according to the method of Ciriminna *et al* [53], pH 4.0. Figure E: ^13^C NMR 100 mM ^13^C_2_-oxalate (Cambridge Isotope Labs), pH 4.0. Figure F: ^13^C NMR of product of the oxidation of ^13^C_3_-glycerol by TEMPO and sodium hypochlorite (66 mM) spiked with 66 mM ^13^C_2_-oxalate (Cambridge Isotope Labs).
(PDF)

## Abbreviations

OxOx: oxalate oxidase
OxDC: oxalate decarboxylase
CsOxOx: OxOx from *Ceriporiopsis subvermispora*
BsOxDC: OxDC from *Bacillus subtilis*
HRP: horseradish peroxidase
ABTS: 2,2’-azinobis-(3-ethylbenzthiazoline-6-sulphonic acid)
