# Supplementary material for: Hydrogen peroxide inhibition of bicupin oxalate oxidase
Source: PLoS One. 2017 May 9;12(5):e0177164. doi: 10.1371/journal.pone.0177164 (PMC5423638; doi:10.1371/journal.pone.0177164)
Supplement: S1 File — Figure A: Standard curve constructed by measuring the ion current (arbitrary scale) at m/z 44 of solutions of known CO2 content. Figure B: The consumption of O2 and production of CO 2 (in arbitrary ion currents) during the CsOxOx catalyzed oxidation of 13C2–oxalate. Figure C: Plot of the ratio of moles CO2 formed per mole of oxygen consumed as a function of oxalate concentration. Text A: The oxidation of 13C3-glycerol by TEMPO and sodium hypochlorite yields 13C2-oxalate. Figure D: 13C NMR of product of the oxidation of 13C3-glycerol by TEMPO and sodium hypochlorite according to the method of Ciriminna et al [53], pH 4.0. Figure E: 13C NMR 100 mM 13C2-oxalate (Cambridge Isotope Labs), pH 4.0. Figure F: 13C NMR of product of the oxidation of 13C3-glycerol by TEMPO and sodium hypochlorite (66 mM) spiked with 66 mM 13C2-oxalate (Cambridge Isotope Labs). (PDF) [file pone.0177164.s001.pdf]

Supporting Information for

Hydrogen Peroxide Inhibition of Bicupin Oxalate Oxidase

John M. Goodwin[1], Hassan Rana[1], Joan Ndungu[1], Gaurab Chakrabarti[2,3,4] and Ellen W. Moomaw[1]*

[1]Department of Chemistry and Biochemistry, Kennesaw State University, Kennesaw, GA.

[2]Department of Pharmacology, Oncology and Radiation Oncology, University of Texas Southwestern Medical Center, Dallas, TX.

[3]Laboratory of Molecular Stress Responses, University of Texas Southwestern Medical Center, Dallas, TX.

[4]Simmons Comprehensive Cancer Center, University of Texas Southwestern Medical Center, Dallas, TX.

* Corresponding author

Email: emoomaw@kennesaw.edu (EWM)

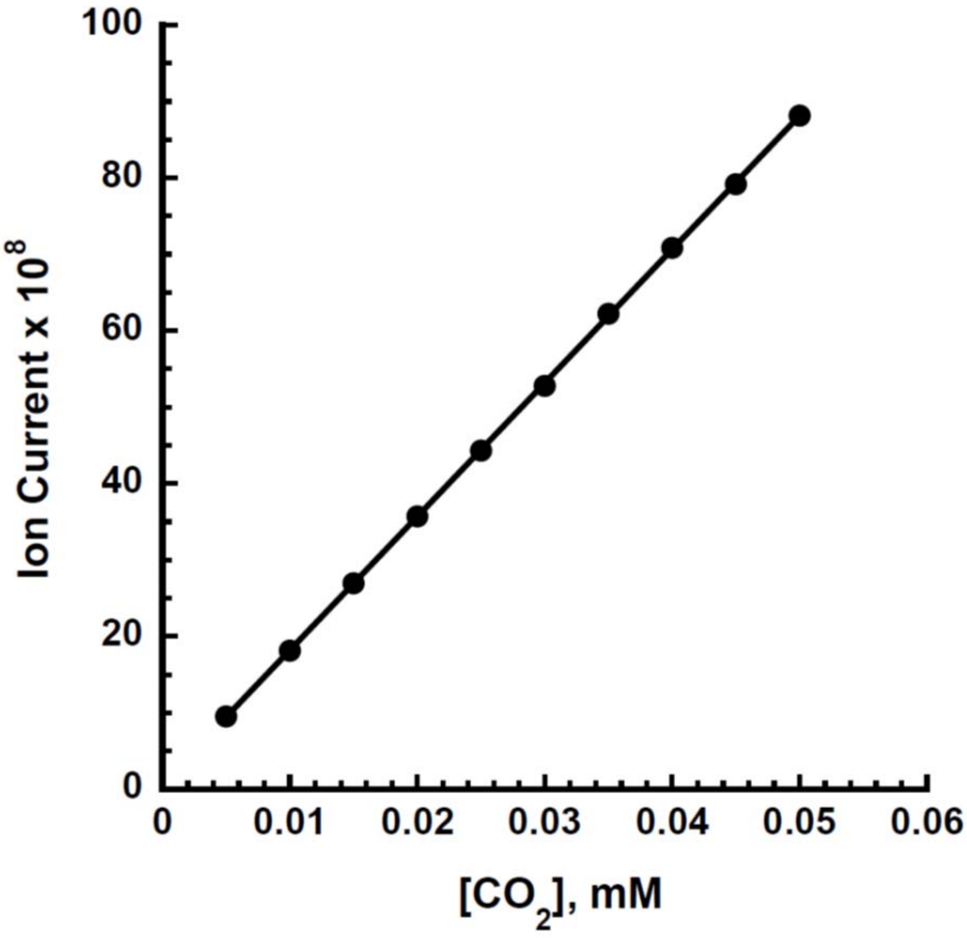

Figure A: Standard curve constructed by measuring the ion current (arbitrary scale) at m/z 44 of solutions of known CO_2 content (prepared as described in the Materials and Methods section) ranging from 0 to 0.05 mM CO_2 in 0.05 mM increments.

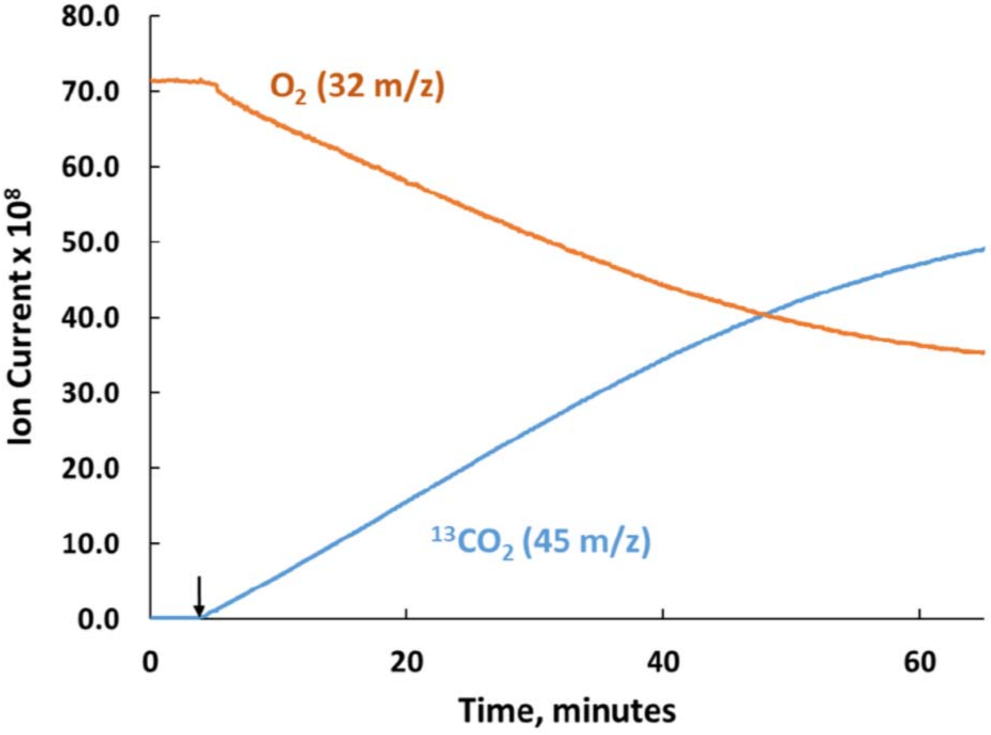

Figure B: The consumption of O_2 and production of CO_2 (in arbitrary ion currents) during the CsOxOx catalyzed oxidation of $^{13}C_2$–oxalate. The ion currents for the dissolved gases at their respective peak heights were recorded: blue, CO_2 at m/z 45; orange, O_2 at m/z 32. The solution contained 10 mM potassium $^{13}C_2$–oxalate in 50 mM sodium succinate buffer at pH 4.0 and 25 ^{o}C. The 2.0 mL reaction was initiated by the addition of recombinant CsOxOx to a final concentration of 0.10 μM at 5 minutes (arrow).

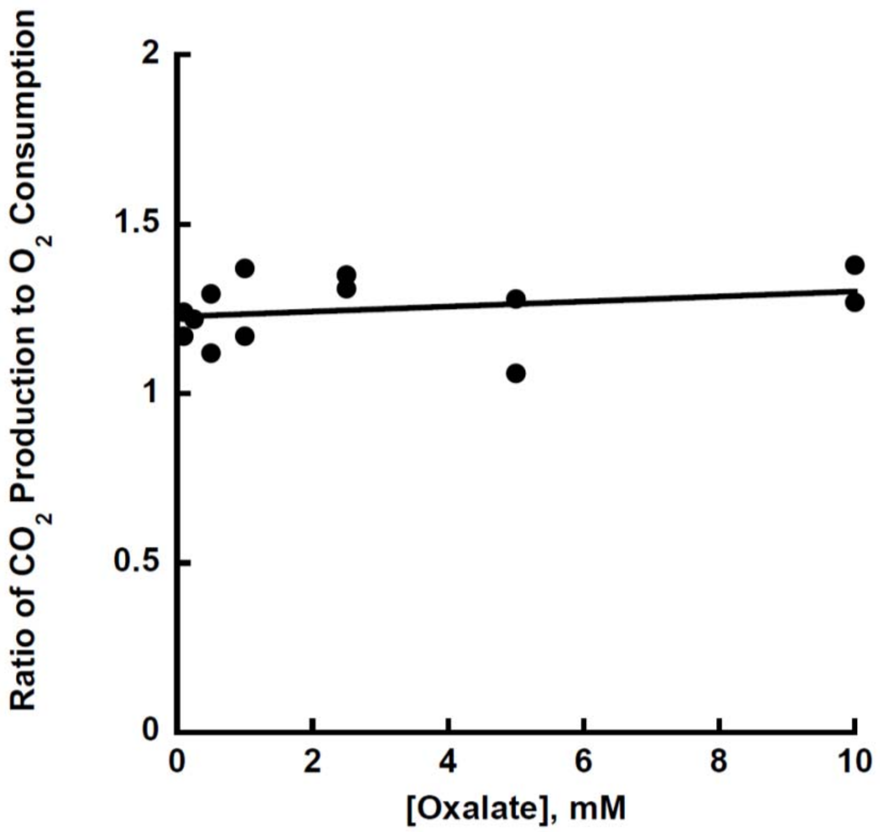

Figure C: The ratio of CO_2 production to O_2 consumption as a function of oxalate concentration.

Text A: The oxidation of $^{13}C_3$-glycerol by TEMPO and sodium hypochlorite yields $^{13}C_2$-oxalate

Preparation and analysis of $^{13}C_2$-oxalate from $^{13}C_3$-glycerol

In an attempt to produce $^{13}C_3$-mesoxalate the oxidation of triply labeled $^{13}C_3$-glycerol was carried out using the small organic catalyst (2,2,6,6-tetramethylpiperidin-1-yl)oxyl (TEMPO) and sodium hypochlorite following the method of Ciriminna *et al* [1]. Since oxalate and mesoxalate are co-elute from the 300mm x 7.8 mm Aminex HPX-87H ion exchange column (Bio-Rad) attached to a Dionex HPLC system as described in the Materials and Methods section, the identity and purity of the product was assessed by ^{13}C NMR. NMR spectra were recorded on a Bruker DPX 300. Chemical shifts are described in parts per million downfield shifted from DMSO. The ^{13}C NMR of the product of the oxidation of $^{13}C_3$-glycerol by TEMPO and sodium hypochlorite prepared according to the method of Ciriminna *et al* [1] at pH 4.0 is shown in Figure S4. The major product is identical that shown in Figure S5 of the ^{13}C NMR 100 mM $^{13}C_2$-oxalate (Cambridge Isotope Labs), pH 4.0. Figure S6 shows the ^{13}C NMR of the product of the oxidation of $^{13}C_3$-glycerol by TEMPO and sodium hypochlorite spiked with $^{13}C_2$-oxalate (Cambridge Isotope Labs) confirming that the product is $^{13}C_2$-oxalate.

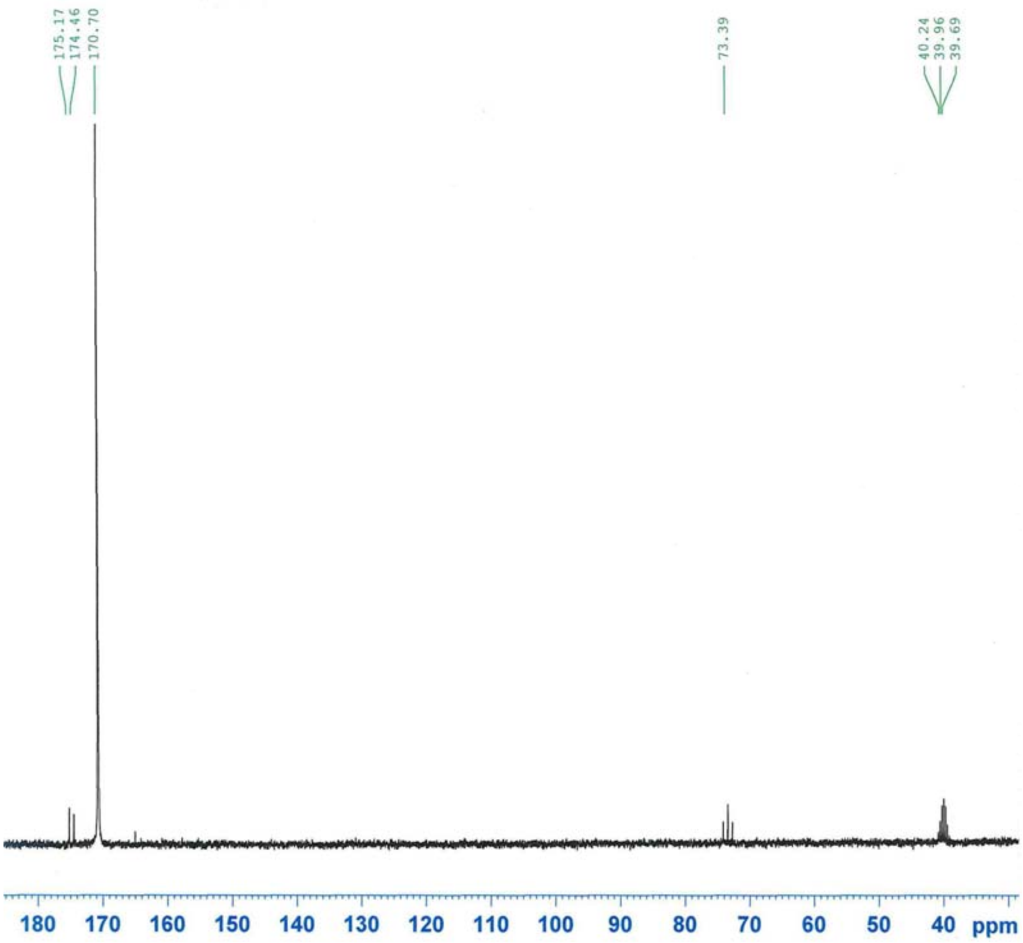

Figure D: ^{13}C NMR of product of the oxidation of $^{13}C_3$-glycerol by TEMPO and sodium hypochlorite according to the method of Ciriminna *et al* [1], pH 4.0.

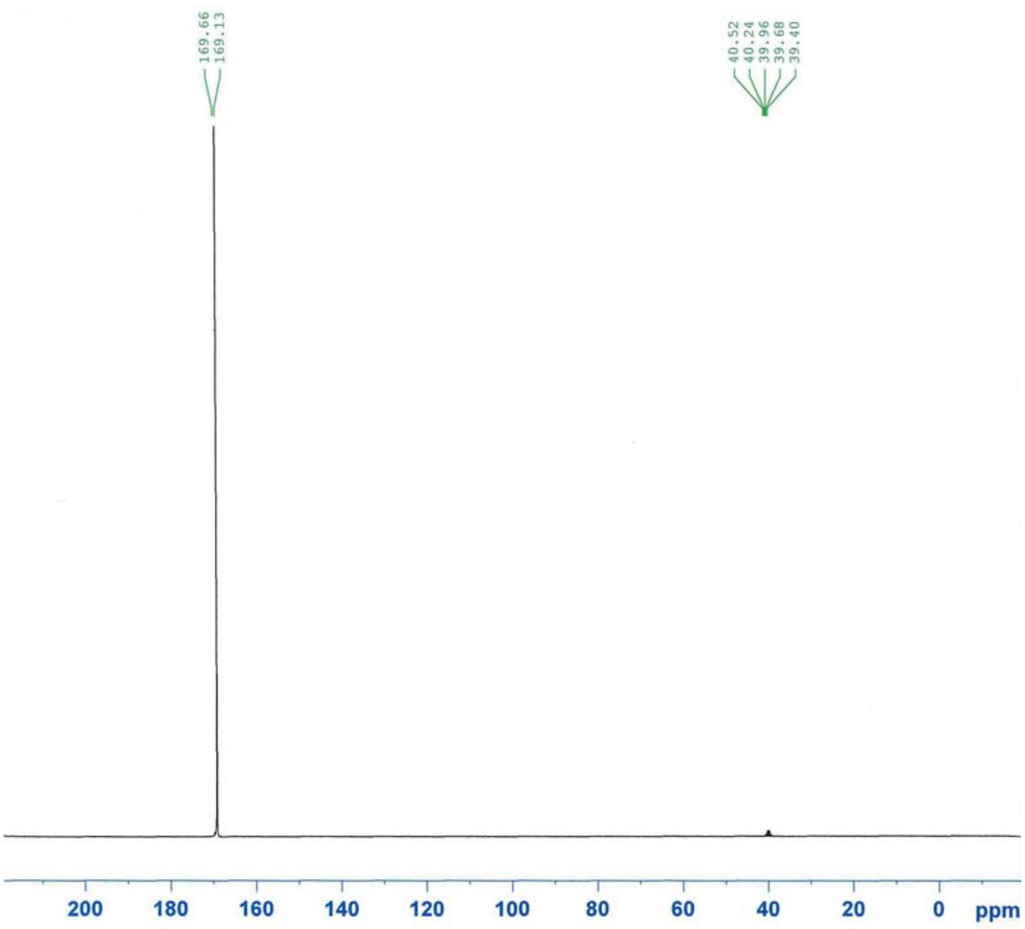

Figure E: ^{13}C NMR 100 mM $^{13}C_2$-oxalate (Cambridge Isotope Labs), pH 4.0.

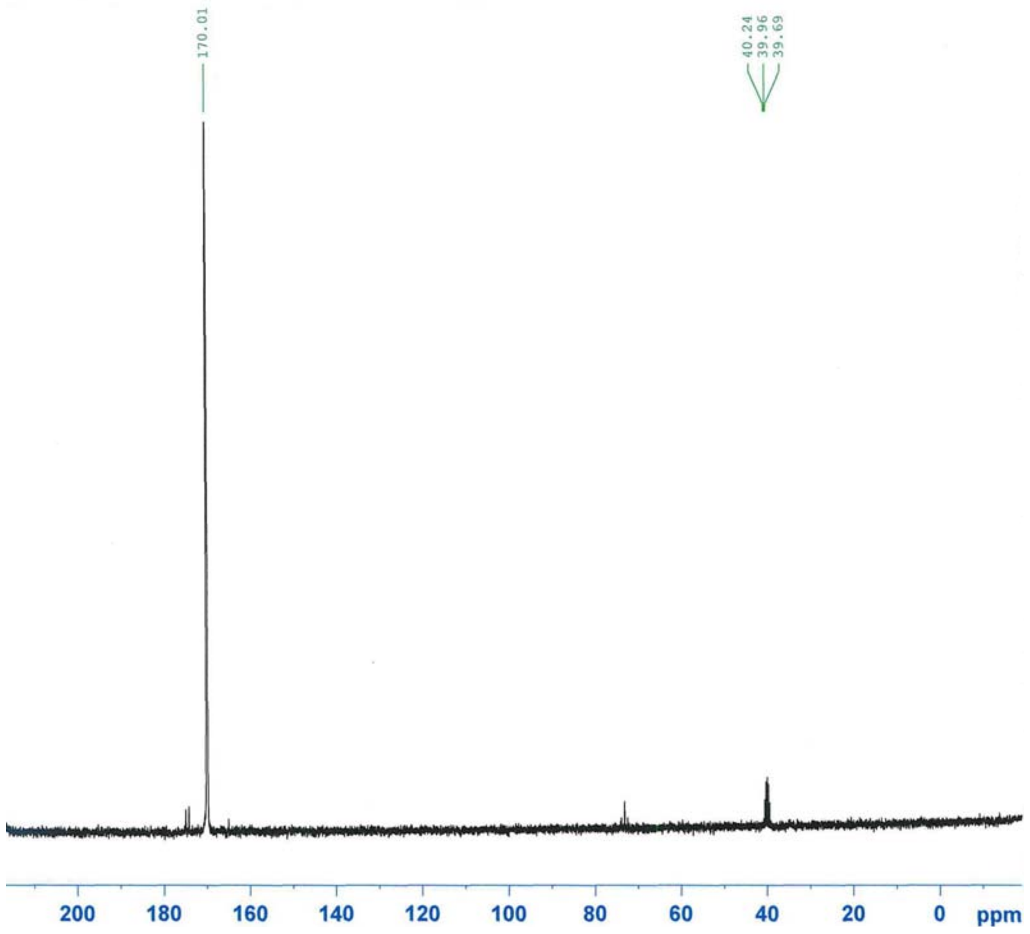

Figure F: ^{13}C NMR of product of the oxidation of $^{13}C_3$-glycerol by TEMPO and sodium hypochlorite (66 mM) with 66 mM $^{13}C_2$-oxalate (Cambridge Isotope Labs).

1. Ciriminna R, Pagliaro M (2003) One-pot homogeneous and heterogeneous oxidation of glycerol to ketomalonic acid mediated by TEMPO. Advanced Synthesis & Catalysis 345: 383-388.
